# The Anti-atherogenic Role of Exercise Is Associated With the Attenuation of Bone Marrow-Derived Macrophage Activation and Migration in Hypercholesterolemic Mice

**DOI:** 10.3389/fphys.2020.599379

**Published:** 2020-11-23

**Authors:** Thiago Rentz, Amarylis C. B. A. Wanschel, Leonardo de Carvalho Moi, Estela Lorza-Gil, Jane C. de Souza, Renata R. dos Santos, Helena C. F. Oliveira

**Affiliations:** ^1^Department of Structural and Functional Biology, Institute of Biology, State University of Campinas, Campinas, Brazil; ^2^Division of Radiotherapy, Faculty of Medical Sciences, Medical School Hospital, State University of Campinas, Campinas, Brazil

**Keywords:** atherosclerosis, exercise, inflammation, macrophages, bone-marrow transplantation

## Abstract

An early event in atherogenesis is the recruitment and infiltration of circulating monocytes and macrophage activation in the subendothelial space. Atherosclerosis subsequently progresses as a unresolved inflammatory disease, particularly in hypercholesterolemic conditions. Although physical exercise training has been a widely accepted strategy to inhibit atherosclerosis, its impact on arterial wall inflammation and macrophage phenotype and function has not yet been directly evaluated. Thus, the aim of this study was to investigate the effects of aerobic exercise training on the inflammatory state of atherosclerotic lesions with a focus on macrophages. Hypercholesterolemic LDL-receptor-deficient male mice were subjected to treadmill training for 8 weeks and fed a high-fat diet. Analyses included plasma lipoprotein and cytokine levels; aortic root staining for lipids (oil red O); macrophages (CD68, MCP1 and IL1β); oxidative (nitrotyrosine and, DHE) and endoplasmic reticulum (GADD) stress markers. Primary bone marrow-derived macrophages (BMDM) were assayed for migration activity, motility phenotype (Rac1 and F-actin) and inflammation-related gene expression. Plasma levels of HDL cholesterol were increased, while levels of proinflammatory cytokines (TNFa, IL1b, and IL6) were markedly reduced in the exercised mice. The exercised mice developed lower levels of lipid content and inflammation in atherosclerotic plaques. Additionally, lesions in the exercised mice had lower levels of oxidative and ER stress markers. BMDM isolated from the exercised mice showed a marked reduction in proinflammatory cytokine gene expression and migratory activity and a disrupted motility phenotype. More importantly, bone marrow from exercised mice transplanted into sedentary mice led to reduced atherosclerosis in the recipient sedentary mice, thus suggesting that epigenetic mechanisms are associated with exercise. Collectively, the presented data indicate that exercise training prevents atherosclerosis by inhibiting bone marrow-derived macrophage recruitment and activation.

## Introduction

Atherosclerosis and resulting cardiovascular diseases are major causes of death worldwide (Libby et al., 2019). Hypercholesterolemia, particularly increased LDL cholesterol, promotes atherosclerosis. The disease is initiated with deposition of cholesterol-rich lipoproteins in the arterial intima, leading to local oxidative stress, activation and recruitment of immune cells and the establishment of an unresolving inflammation process (Koelwyn et al., 2018; Mury et al., 2018). Macrophages play crucial roles in all phases of atherosclerosis, including the initiation and progression to advanced lesions. Their content and presence at injury sites reflects their capacity for differentiation, proliferation, retention, emigration and death of both resident and macrophages from blood-borne monocytes (Moore et al., 2018). Driven by chemotaxis and differentiation factors, the majority of monocytes newly recruited from bone marrow and other medullary organs become cells with macrophage-like features that engulf the retained and modified lipoproteins, a process that is initially favorable but ultimately results in the accumulation of lipid-filled foam cells that trigger plaque formation (Moore et al., 2018; Xu et al., 2019). Foam cell development is usually associated with a classical M1 proinflammatory phenotype (Chinetti-Gbaguidi et al., 2011), which is critical for a well-characterized inflammatory response that includes secreting interleukins and chemokines and being a major source of large amounts of oxidants species, such as those derived from NADPH oxidase activation and inducible nitric oxide synthase (iNOS) (Tabas and Bornfeldt, 2016). Phenotypic and functional alterations of macrophages in lesions have profound consequences for plaque biology (Leitinger and Schulman, 2013; Tabas and Bornfeldt, 2016). For instance, M1 macrophages seems to be the predominant phenotype in rupture prone shoulder regions of the plaques while the M2 anti-inflammatory macrophages are predominant in the adventitia (Stöger et al., 2012). Chinetti-Gbaguidi et al. (2011) have localized M2 macrophages in more stable cell-rich areas of plaque away from the lipid core. On the other hand, several other macrophage populations, distinct from the M1 and M2 extremes, likely evoked by the microenvironment stimuli and activation of specific intracellular signaling pathways are present in atherosclerotic lesions, representing a wide spectrum of phenotypes and functions. These macrophages play key roles in lesion initiation, progression, necrosis, remodeling, regression, and resolution (Tabas and Bornfeldt, 2016).

Changes in lifestyle, including diet and regular physical exercise, have been widely accepted as powerful strategies to decrease the risks of developing atherosclerosis and induce its amelioration (Adams et al., 2017; Fiuza-Luces et al., 2018). Numerous studies on humans have shown cardiovascular risk improvements achieved through different types of physical exercise training, such as improved lipid and lipoprotein profiles, glycemic control, endothelial function, and antioxidant status (Palmefors et al., 2014). However, data on the direct anti-atherogenic effects of physical exercise in the general population are scarce. Rauramaa et al. (2004) found in a randomized, controlled trial, excluding men taking statins, that the progression of intima-media thickness in the carotid was 40% less in the exercised group compared to the control group in a 6-year period. In experimental studies, more abundant data on the relationship between physical exercise and atherosclerosis were found. Exercise slows the progression of atherosclerosis in hyperlipidemic animal models, such as ApoE^–/–^ and LDLr^–/–^ mice (Ramachandran et al., 2005; Shimada et al., 2007; Guizoni et al., 2016; Jakic et al., 2019), promotes stabilization and prevents plaque rupture (Pynn et al., 2004; Napoli et al., 2006; Pellegrin et al., 2009; Kadoglou et al., 2011), whereas physical inactivity accelerates atherosclerosis development (Laufs et al., 2005; Mury et al., 2018). The positive effects of physical exercise are attributed in part to general antioxidant action, alleviation of endoplasmic reticulum (ER) stress-mediated endothelial dysfunction and local oxidative stress (Okabe et al., 2007; Hong et al., 2018) and promotion of anti-inflammatory action (Chen et al., 2010; Kawanishi et al., 2010). Accordingly, exercise training lowers the levels of circulating inflammatory monocytes (CD14^+^ CD16^+^) in humans (Timmerman et al., 2008). In addition, exercise training, through promoting shear stress and mechanical stimulus, increases endothelial nitric oxide (NO) synthase (eNOS) expression and NO production in the vasculature. Because NO has numerous anti-atherosclerotic properties, increased eNOS expression in response to exercise can explain part of the beneficial effects of exercise in cardiovascular disease (Harrison et al., 2006). In an experimental model of vascular disease, ApoE^–/–^ mice supplemented with the cofactor BH4 had increased eNOS activity and NO bioavailability and markedly reduced infiltration of T-cells, macrophages and monocytes into plaques, and reduced T-cell infiltration in the adjacent adventitia (Schmidt et al., 2010).

Although in-depth knowledge about the overall anti-atherogenic role of physical exercise has been attained, data concerning the impact of exercise directly on macrophage phenotype and function, which are causally linked to arterial wall lesion development, remain scarce. Therefore, here, we investigate the potential of physical exercise training to modify concurrent (*in vivo* and *ex vivo*) and long-lasting (bone marrow transplantation) macrophage features relevant to the atherosclerosis context. We subjected hypercholesterolemic mice lacking the LDL receptor (LDLr^–/–^) to a program of moderate aerobic treadmill training and an atherogenic diet. Subsequently, we evaluated 1- lipid load, inflammatory macrophage content and oxidative stress markers of atherosclerotic plaques; 2- gene expression, migratory activity and cytoskeleton organization of bone marrow-derived macrophages; and 3- the capacity of bone marrow from exercised mice to reduce atherosclerosis in sedentary mice.

## Materials and Methods

### Animals

Low-density lipoprotein receptor-knockout [LDLr^–/–^ (B6. 129S7-Ldlrtm1Her/J)] male and female mice with a C57BL6/J background, originally from the Jackson Laboratory (Bar Harbor, ME), were obtained from the breeding colony at the State University of Campinas (UNICAMP) and maintained under controlled temperature (22 ± 1°C) in a 12 h dark/light cycle in a local (conventional) animal facility in individually ventilated cages (4–5 mice/cage), with unlimited access to filtered water and regular rodent AIN93-M diet (Supplementary Table 1). All animal experiments were performed in the Laboratory of Lipid Metabolism in the Department of Structural and Functional Biology, Institute of Biology, State University of Campinas. The experimental protocols were approved by the University Committee for Ethics in Animal Experimentation (CEUA/UNICAMP #3002-1) and were performed in accordance with national Brazilian guideline number 13 for “Control in Animal Experiments,” published on September 13th, 2013 (code 00012013092600005, available at: http://portal.in.gov.br/verificacao-autenticidade). Procedures adhered to ARRIVE (Animal Research: Reporting of *In Vivo* Experiments) guidelines.

### Training Protocol

Male LDLr^–/–^ mice (8 weeks old) were randomly assigned to an exercise training (Exe) or sedentary (Sed) group, and both mouse groups were fed a high-fat diet (35 g% fat, Supplementary Table 1) (Pragsoluções Biociências, Jaú, SP, Brazil) for 8 weeks. Before the training program, at 7 weeks of age, the mice were familiarized with the treadmill by undergoing five sessions of 15 min of treadmill (10 cm/sec) for 1 week. The exercise training protocol consisted of a 1-h session on a treadmill, five times a week, at a 45° slope, and 50–60% VO_2_max for 8 weeks. The speed corresponding to the 50–60% VO_2_max was determined after the maximal exercise test, described below. The mouse control group remained sedentary. Before starting the training period, mice were submitted to a maximal (incremental) exercise test on an inclined treadmill (45° slope) to determine the speed corresponding to the VO_2_max. The test consisted of 5 min at 10 cm/s, followed by increases of 5 cm/s every min until exhaustion, which was defined as the point at which mice stopped running or touched the end of the treadmill five times in 1 min. This test provided data on the exhaustion time, distance run and the peak workload (maximal speed/VO_2_max) (Guizoni et al., 2016). The maximal speed at the beginning of the protocol was ∼22 cm/s for both groups (Supplementary Figure 1). Based on this test, an initial training speed of 13 cm/s (corresponding to 50–60% of the maximal speed) was applied to the exercise training group, with speed increments of 1 cm/s per week during the 8 weeks of training (final week speed of 20 cm/s). At the end of the physical exercise program, the effectiveness of the training was evaluated again by the same incremental exercise test on the treadmill for both the sedentary and exercised mice. As expected, after 8 weeks of training, we observed significant increases in exhaustion time (44%), distance run (84%), and maximal speed (47%) in the exercised LDLr^–/–^ mice compared with the sedentary LDLr^–/–^ mice, while no differences between the groups were observed before the training period (Supplementary Figure 1).

### Lipid, Lipoprotein, Glucose, and Cytokine Measurements

Blood was collected via the retro-orbital plexus of anesthetized mice for plasma analyses. Total cholesterol and triglyceride (TG) concentrations were measured using colorimetric-enzymatic assays (Chod-Pap; Roche Diagnostic GmbH, Mannheim, Germany). The cholesterol distribution in plasma lipoprotein fractions was determined by fast protein liquid chromatography (FPLC) gel filtration with Superose 6 HR 10/30 columns (Pharmacia) and subsequent cholesterol determination of the collected fractions. Blood glucose concentrations were measured using a glucose analyzer (Accu-Chek Advantage, Roche Diagnostic, Switzerland). IL-1β, IL-6, IL-10, and TNF-α plasma concentrations were determined by ELISA (R&D Systems, Minneapolis, United States).

### Aortic Root Histology and Immunostaining

At the end of the experimental period, the mice were anesthetized with xylazine/ketamine (10 and 50 mg/kg, respectively, *ip*). Mouse hearts were perfused with 10 mL of phosphate-buffered saline (PBS) and fixed overnight in 10 mL of 4% paraformaldehyde (PFA). After incubation in PFA, the hearts were washed with PBS and left in PBS for 1 h. Next, the hearts were embedded in OCT compound (Sakura Inc., Torrance, CA, United States) and frozen at −80°C. The tip of the heart (ventricle) was removed with a surgical knife. Serial slices of 60 μm were cut using a cryostat and discarded until the aortic sinus leaflets were visible. Then, the slices were cut to a 10-μm thickness; two sliced sections were placed on separate slides until a total aorta length of 640 μm was reached. These sliced sections were stained with oil red O. The red areas of the lesions were calculated as the sum of lipid-stained lesions. The lipid stained lesions were quantified using ImageJ (1.45 h) software. An investigator who was unaware of the treatments evaluated the slides. The same procedures of cryo-sectioning were employed on other hearts that were used for immunofluorescence staining of CD68, CD68 + IL-1β, CD68 + GADD153, nitrotyrosine (3-NT) and MCP-1. The sections were blocked with 10% bovine serum albumin (BSA) and then incubated for 3 h at 22°C (RT) or overnight at 4°C with the following primary antibodies: rat anti-CD68 (1:250; AbD Serotec), goat anti-IL-1β (1:50; Santa Cruz Biotechnology), rabbit anti-GADD153 (1:50; Santa Cruz Biotechnology), rat anti-MCP-1 (1:150; Abcam), and biotinylated nitrotyrosine (3-NT) (1:200; Cayman Chemical). The sections were washed and incubated with fluorescent Alexa Fluor-conjugated secondary antibody (Invitrogen). Nuclei were counterstained for 10 min with DAPI. The sections were mounted with VECTASHIELD medium, and pictures were taken with a Leica DMI600B microscope using a 20× objective. Microscopic images of the aortic root sections were digitized, and morphometric measurements were recorded. ImageJ software (NIH-ImageJ, United States) was used for all quantification procedures.

### Reactive Oxygen Species (ROS) Measurement

*In situ* ROS production was estimated using the oxidation of the dihydroethidium (DHE) probe to fluorescent products (Davel et al., 2012). Transverse aortic sections (10 μm) obtained with a cryostat were incubated with PBS at 37°C for 10 min. Fresh PBS containing DHE (5 μM) was topically applied to each tissue section, and the slices were incubated in a light-protected humidified chamber at 37°C for 30 min and then a coverslip was added. Negative control sections were treated with the same volume of PBS as the experimental section but without DHE. Images were obtained with a Leica DMI600B microscope equipped for epifluorescence detection using a 20× objective. ImageJ software (NIH-ImageJ, United States) was used for the quantification.

### Bone Marrow-Derived Macrophage (BMDM) Isolation

Bone marrow was aseptically flushed from the tibias and femurs with DMEM high glucose (4.5 g/L glucose) (Vitrocell) and 10% fetal bovine serum (FBS) and centrifuged at room temperature for 5 min at 1,000 rpm. Then, the cells were resuspended in 2 mL of red blood cell-lysing buffer (Sigma) and incubated for 5 min for the complete lysis of the red blood cells and preservation and concentration of nucleated white blood cells. Two milliliters of sterile PBS was added to neutralize the lytic reaction. The cells were centrifuged for 5 min at room temperature and resuspended in complete DMEM supplemented with 15% L929 cell-conditioned media, 10% FBS and 1% penicillin/streptomycin. Next, these cells were plated and cultured in complete medium for 7 days to induce macrophage differentiation (BMDM). On the fourth day of culture, contaminating non-adherent cells were eliminated by changing one-half of the medium. On the eighth day, the culture supernatants were discarded. The remaining adherent cells were washed with 5 mL of pre-warmed PBS. The PBS washes were discarded, and the adherent cells were gently scraped to detach them in preparation for the migration assay, immunofluorescence staining and RNA extraction.

### BMDM Migration Assay

Cell migration assays were performed with a 24-well Boyden chamber using Costar Transwell inserts with a 8-μm pore size (Corning) as previously described (Rotllan et al., 2015). Under sterile conditions, BMDM were incubated in serum-free DMEM at 37°C and 5% CO_2_ for 1 h prior to the assay. The chemoattractant MCP-1 (R&D Systems), prepared to 100 ng/mL in serum-free DMEM was added to the bottom chambers. BMDM were then carefully added to the top of the filter membrane of the Transwell insert (upper chamber) (2.5 × 10^5^ cells/mL) and incubated at 37°C and 5% CO_2_ for 4 h. Then, the Transwell inserts were removed, and the BMDMs were fixed with 4% paraformaldehyde in PBS for 5 min, washed with PBS twice and stained with crystal violet (0.1%, Invitrogen) for 15 min at room temperature. The cells remaining on the top of the membrane (inner side of the insert) were gently removed with a cotton swab. An inverted Olympus BX51 microscope connected to an Olympus DP72 digital camera (10× objectives) was used to obtain images of the cells attached to the outer side of the insert membrane. Cell numbers represent the average of counts of five random visual fields. Experiments were performed in triplicate; the results represent four independent experiments.

### BMDM Immunofluorescence Staining and Confocal Imaging

BMDM were seeded onto 8-well Lab-Tek slides (Thermo Fisher Scientific) using macrophage growth medium. These cells were serum-starved for 1 h and then stimulated with 100 ng/mL MCP-1 (R&D System) for 5 and 15 min. These BMDM were fixed with 4% PFA in PBS for 10 min at room temperature. Then, these cells were permeabilized with 0.2% Triton X-100 in PBS for 5 min and stained with anti-RAC-1 (1:100, Sigma). The cells were then incubated with fluorescent secondary antibody [Alexa Fluor 488 anti-mouse (Invitrogen)]. F-actin was visualized with rhodamine-conjugated phalloidin (Invitrogen). Nuclei were counterstained with DAPI (Sigma) for 10 min. Finally, the cells were mounted onto glass slides with VECTASHIELD mounting medium (Vector Laboratories). Images of the cells were acquired using a Leica LSM 780 confocal microscope with a 63× objective, followed by measurement of the average cell areas of at least 10 cells per field using ImageJ software. The results are representative of at least three independent experiments.

### Real-Time PCR

BMDM mRNA was extracted using an RNeasy kit (Qiagen, Cat. #74007). One microgram of purified mRNA was used to synthesize cDNA (high-capacity cDNA reverse transcription kit, Applied Biosystems, Foster City, CA). Relative quantification was performed using the StepOne real-time PCR system (Applied Biosystems). The primers were designed and tested against the *Mus musculus* genome (GenBank). The relative quantities of the target transcripts were calculated from duplicate samples (2ΔΔCT), and the data were normalized against the endogenous control GAPDH. The studied genes were CD36, IL-1β, MCP-1, IL-6, TNF-α, SOD1, GADD153, and CDC42. The primer sequences are shown in Supplementary Table 2.

### Irradiation and Bone Marrow Transplantation

For bone marrow transplantation we chose female LDLr^–/–^ mice as graft recipient, since they exhibit more and larger lesions than males. We also chose to use a cholesterol containing high fat diet to induce more severe atherosclerosis (22 g% and 0.15 g% of fat and cholesterol, respectively) (Pragsoluções Biociências, Jaú, SP, Brazil) (Supplementary Table 1). Male LDLr^–/–^ donor mice were subjected to a 16-week treadmill training or remained sedentary and fed a high-fat diet. Seven-week-old female LDLr^–/–^ recipient mice were exposed to a single 8.0-Gy total-body irradiation dose using a 6 MV linear accelerator (Clinac 2100C, Varian Medical Systems, United States). The donor bone marrow cells were aseptically harvested by flushing the femurs and tibias from male LDLr^–/–^ sedentary or exercised mice with Dulbecco’s PBS containing 2% fetal bovine serum. The samples were filtered through a 40 μm nylon mesh and centrifuged at room temperature for 10 min at 1,000 rpm. The cells were resuspended in Dulbecco’s PBS containing 2% fetal bovine serum to a concentration of 5.0 × 10^6^ viable nucleated cells in 200 μL for use in each recipient mouse. Female LDLr^–/–^ mice were randomly assigned to groups to receive bone marrow from the exercised LDLr^–/–^ or sedentary LDLr^–/–^ mice via intravenous injection (tail vein). Subsequently, the recipient mice were housed in sterilized cages and treated with antibiotics (0.2 mg Bactrim^®^ trimethoprim and 1.0 mg/mL sulfamethoxazole) in the drinking water for 4 days before and 7 days after bone marrow transplantation. After 1 week of recovery, the mice had free access to sterile water and a sterile high-fat and high-cholesterol diet for 8 weeks. For terminal experiments, mice were anesthetized with xylazine/ketamine (10 and 50 mg/kg, respectively, *ip*), and hearts were perfused and excised for atherosclerosis and macrophage content analysis.

### Statistical Analysis

The data are presented as the means ± the standard error (SE) and (n) is provided in each figure legend. Two mean comparisons were evaluated with two tailed Student’s *t*-test. Significance was accepted at the level of *p* ≤ 0.05.

## Results

### Exercise Training Reduces Body and Fat Mass, Increases HDL Cholesterol and Decreases Inflammatory Cytokine Plasma Levels

Body and fat mass and food/water consumption after chronic moderate aerobic training are shown in Table 1. The exercise protocol resulted in a reduction in body weight (10%) and epididymal fat pad (40%). No differences in food intake were observed. The sedentary LDLr^–/–^ group gained weight and exhibited greater accumulation of adipose tissue than their exercised counterparts despite similar levels of food consumption. Water consumption was 27% higher in the exercised LDLr^–/–^ mice than in the sedentary mice. The blood biochemistry parameter measurements are shown in Table 2. The plasma glucose levels did not differ between the sedentary and exercised LDLr^–/–^ mice, while the plasma triglyceride levels were 16% lower in the exercised group. Total plasma cholesterol levels were similar in both groups. However, the FPLC lipoprotein fraction showed significantly increased HDL cholesterol in the exercised LDLr^–/–^ mice (Table 2). Circulating levels of inflammatory cytokines were significantly diminished in the exercised LDLr^–/–^ mice as follows: 48% for IL-6, 77% for TNF-α, and 76% for IL-1β (Table 2). These results demonstrate that aerobic exercise training was effective in counteracting diet-induced systemic inflammation.

**TABLE 1:** Food and water consumption, body and tissue weights of the sedentary and exercised LDLr^–/–^ mice fed a high-fat diet for 8 weeks.

	LDLr^–/–^ Sed	LDLr^–/–^ Exe
Body weight (g)	27.65 ± 0.74 (15)	24.92 ± 0.49 (15)**
Epididymal fat pad (g/100 g)	0.997 ± 0.09 (15)	0.588 ± 0.06 (15)**
Brown fat (g/100 g)	0.105 ± 0.003 (15)	0.093 ± 0.004 (15)
Liver (g/100 g)	0.859 ± 0.01 (15)	0.833 ± 0.01 (15)
Food intake (g/week)	17.01 ± 1.13 (15)	14.60 ± 0.40 (15)
Water consumption (mL/week)	23.10 ± 1.87 (15)	29.45 ± 1.52 (15)*

Data are the mean ± SE (n). *P < 0.05, **P < 0.01. Student’s t-test.

**TABLE 2:** Plasma biochemical profile of the sedentary and exercised LDLr^–/–^ mice fed a high-fat diet for 8 weeks.

	LDLr^–/–^ Sed	LDLr^–/–^ Exe
Glucose (mg/dL)	96.40 ± 3.36 (10)	94.30 ± 3.57 (10)
Triglycerides (mg/dL)	123.7 ± 5.63 (15)	102.9 ± 4.38 (15)**
Total cholesterol (mg/dL)	452.3 ± 28.78 (15)	413.1 ± 30.66 (15)
VLDL cholesterol (%)	4.9 ± 1.4 (4)	3.3 ± 0.6 (4)
IDL + LDL cholesterol (%)	62.8 ± 2.7 (4)	53.7 ± 2.9 (4)
HDL cholesterol (%)	31.7 ± 3.1 (4)	42.6 ± 2.9 (4)*
**Cytokines**		
Interleukin-1β (pg/mL)	23.2 ± 5.6 (5)	5.5 ± 2.6 (5)*
Interleukin-6 (pg/mL)	4.3 ± 0.4 (5)	2.2 ± 0.3 (6)**
Tumor necrosis factor-α (pg/mL)	31.1 ± 8.3 (5)	7.2 ± 0.84 (5)*

Data are the mean ± SE (n). *P < 0.05, **P < 0.01. Student’s t-test.

### Exercise Training Decreases Lipid Deposition and Inflammation in the Atherosclerotic Lesions

**FIGURE 1:**
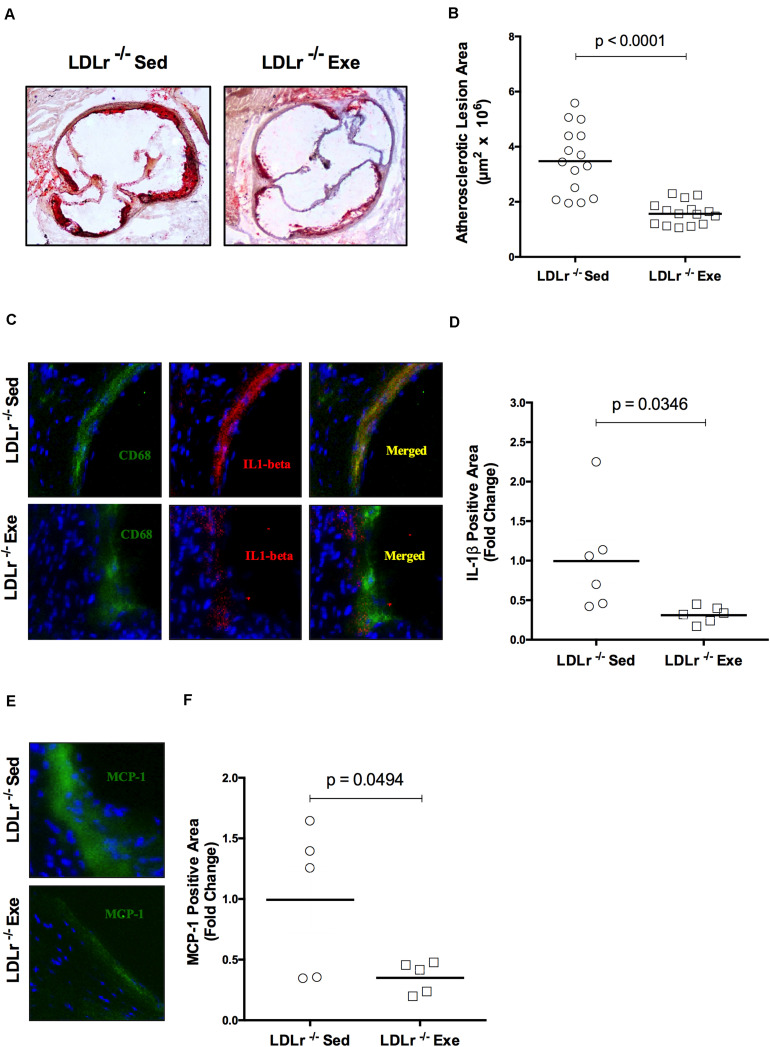
Exercise training decreases the lipid level and attenuates the inflammation in aortic root atherosclerotic lesions. Comparison of sedentary (Sed) and exercised (Exe) LDLr^–/–^ mice fed a high-fat diet for 8 weeks. The lipid stained (oil red O) representative images **(A)** and lesion areas (*n* = 15) **(B)**. Immunofluorescence staining (*n* = 5–6) for macrophage CD68 (green) and IL-1β (red) and merged images of CD68 and IL-1β (yellow) **(C)** and IL-1β positive areas **(D)**; immunofluorescence staining for MCP-1/CCL2 (green), DAPI for nuclei (blue) **(E)**, and MCP-1 positive areas **(F)**. Data are presented as the means and individual determinations. *P*-values according to the Student’s *t*-test.

Concerning artery atherosclerotic lesions, exercise training induced a marked reduction in the lipid-stained plaque area in the aortic root of the exercised LDLr^–/–^ mice (55%) compared with that in the sedentary LDLr^–/–^ group (Figures 1A,B). To analyze lesion inflammatory status, we performed immunofluorescence double staining for macrophages (CD68) and IL-1β (Figure 1C). We observed lower levels of IL-1β (68%) in the arteries of the exercised mice (Figures 1C,D). In addition, the amount of aortic sinus monocyte chemoattractant protein-1 (MCP-1), a major inflammatory chemokine, was reduced by 64% in the lesions of the exercised LDLr^–/–^ mice (Figures 1E,F). These findings suggest that physical exercise reduces macrophage infiltration and activation.

### Exercise Training Decreases Aortic Oxidative and Nitro-Oxidative Damage and ER Stress Markers

**FIGURE 2:**
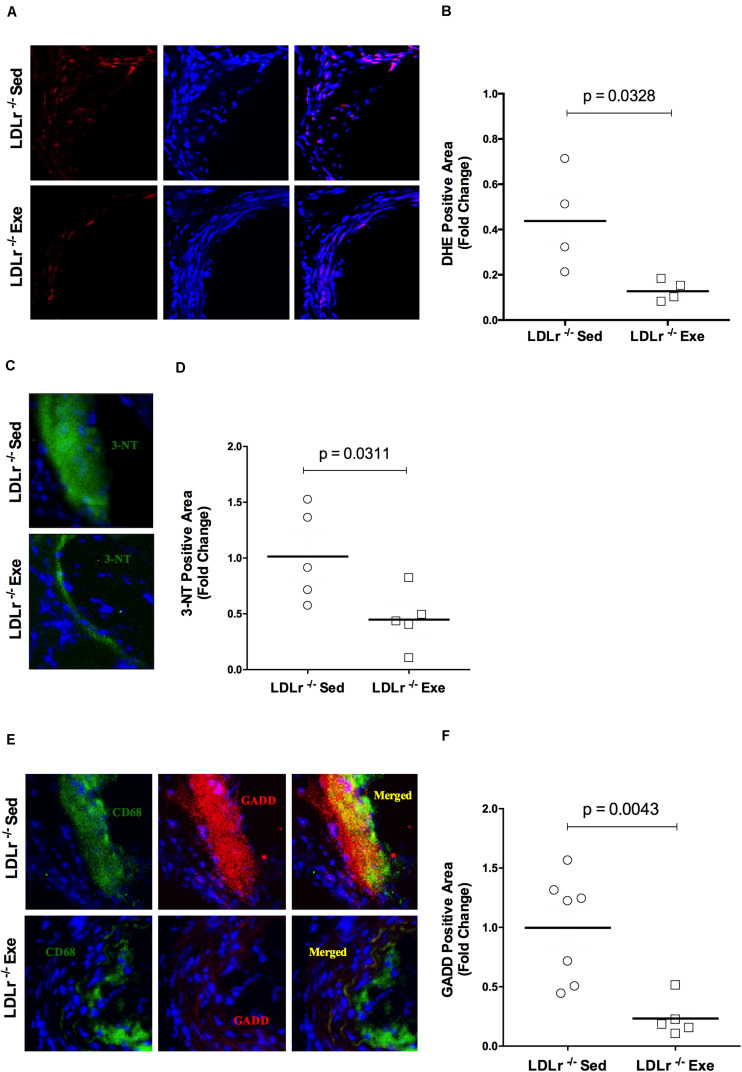
Exercise training reduces oxidative, nitro-oxidative and ER stress in atherosclerotic lesions. Comparison of sedentary (Sed) and exercised (Exe) LDLr^–/–^ mice fed a high-fat diet for 8 weeks. Representative images and quantitative fluorescence analyses of sections stained with the probe dihydroethidium (DHE, red) **(A,B)**, nitrotyrosine antibody (3-NT, green) **(C,D)**, CD68 antibody (for macrophages, green), GADD153 antibody (red) and merged images (yellow) **(E,F)**. Blue represents nuclei stained with DAPI. Data are presented as the means and individual determinations (*n* = 4–7). *P*-values according to the Student’s *t*-test.

Examining the aortic root for oxidative stress, we found that exercise training promoted a reduction in the fluorescence of the reactive oxygen species (ROS) sensitive DHE probe in the atherosclerotic plaques of the exercised LDLr^–/–^ mice. As shown in Figures 2A,B, physical exercise markedly inhibited DHE fluorescence (reduction of 70%), suggesting the inhibition of local ROS production. In addition, nitro-oxidative stress was evaluated by using a nitrotyrosine antibody. Figures 2C,D shows the marked decrease in nitrotyrosine staining of the aortic lesions in the exercised LDLr^–/–^ group (55%), indicating that physical exercise also prevented the formation of nitrated proteins. Moreover, endoplasmic reticulum (ER) stress, which is known to occur during diet-induced atherogenesis, was evaluated by staining the aortic sinus for GADD153 (growth arrest DNA damage protein, also known as CHOP, CCAAT-enhancer-binding protein homologous protein), an undetectable protein in the absence of ER stress. We observed that physical exercise suppressed GADD153 expression in the aortic roots of the exercised LDLr^–/–^ mice by 76% compared to the abundant GADD153 expression and colocalization with macrophages observed in the aortas of the sedentary LDLr^–/–^ mice (Figures 2E,F).

### Exercise Training Attenuates Migratory Activity, Cytoskeletal Motility Organization and Inflammatory Gene Expression in Bone Marrow-Derived Macrophages (BMDM)

**FIGURE 3:**
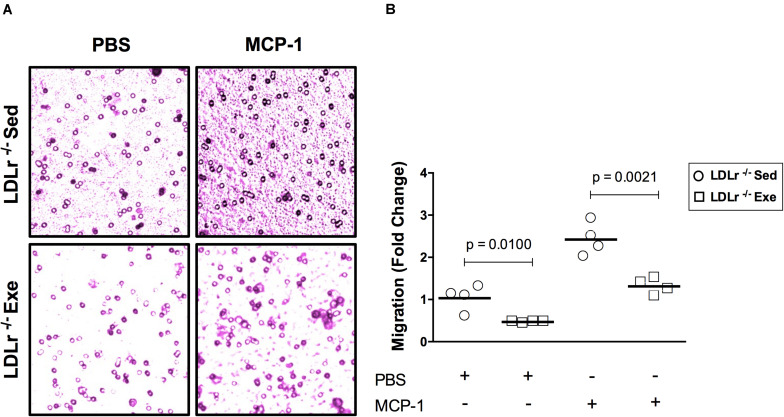
Exercise training reduces the migratory activity of bone marrow-derived macrophages (BMDM) in response to MCP-1. Migration of the BMDM of the sedentary and exercised LDLr^–/–^ mice fed a high-fat diet for 8 weeks after incubation with serum-free DMEM (basal condition) and MCP-1 (100 ng/mL, stimulated condition) for 4 h. At the end of the incubation period, the cells were stained with crystal violet **(A)** and counted **(B)**. Data are presented as the means and individual determinations of four independent experiments performed in triplicate. *P*-values according to the Student’s *t*-test.

An initial event in atherogenesis is the recruitment of monocytes to susceptible sites of the vascular wall by monocyte chemotactic protein-1 (MCP-1). Therefore, we evaluated the migration response of the BMDM from both sedentary and exercised LDLr^–/–^ mice toward MCP-1 (Figure 3). Compared to the basal state, the addition of MCP-1 significantly increased BMDM migration, by twofold in both groups. However, it is noteworthy that in both the basal and stimulated conditions, a 40–50% reduction in migratory activity was observed in the BMDM from the exercised mice (Figures 3A,B).

**FIGURE 4:**
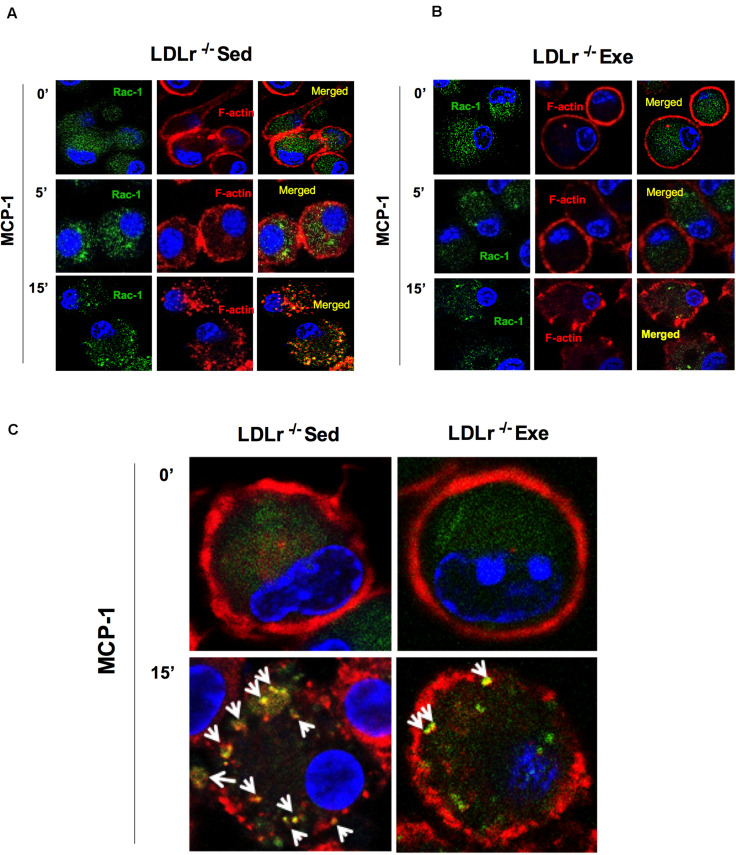
Exercise training disrupts the motility phenotype of bone marrow-derived macrophages (BMDM) in response to MCP-1. Motility phenotype of the BMDM of the sedentary and exercised LDLr^–/–^ mice fed a high-fat diet for 8 weeks as indicated by immunostaining for Rac-1 and F-actin. The cells were treated with MCP-1 (100 ng/mL) after 1 h of serum starvation. At the indicated time points, the cells were fixed and stained for Rac-1 (green) and F-actin (phalloidin, red) and with DAPI (blue). Representative confocal microscopy images are shown at the original magnification of 63× **(A,B)** and with a 4× zoom objective **(C)**. Images representative of three independent experiments.

To confirm the effect of physical exercise in inhibiting the chemotactic response of macrophages, we measured changes in the organization of the cytoskeleton necessary for cell motility. RAC-1 is a protein involved in a variety of cytoskeleton remodeling processes in different cell types. It induces actin polymerization and lamellipodia formation. Stimulation of the BMDM with MCP-1 induced a marked reorganization of the cytoskeleton, as shown by the increase in the amount of RAC-1, disruption of the surface localization of F-actin and colocalization of RAC-1 with F-actin in the BMDM from sedentary LDLr^–/–^ mice (Figures 4A,C). This effect was profoundly attenuated in the cells from the exercised LDLr^–/–^ mice (Figures 4B,C).

**FIGURE 5:**
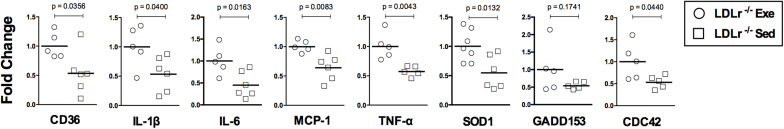
Exercise training downregulates the expression of genes involved in lipid uptake (CD36), inflammation (IL-1β, IL-6, MCP-1, and TNFα), oxidative stress (SOD) and motility (CDC42) in bone marrow-derived macrophages (BMDM). Gene expression (normalized by GAPDH) in the BMDM of the sedentary (Sed) and exercised (Exe) LDLr^–/–^ mice fed a high-fat diet for 8 weeks. The expression of the ER stress marker GADD153 did not vary significantly between groups. Data are presented as the means and individual determinations (*n* = 5–7). *P*-values according to the Student’s *t*-test.

To confirm previous results, we investigated the expression of genes encoding key proteins involved in lipid and lipoprotein uptake (CD36), inflammatory polarization phenotype (IL-1β, IL-6, MCP-1, and TNFα), oxidative stress (SOD1), ER stress (GADD153) and migration/motility activity (CDC42) in the BMDM (Figure 5). We observed that aerobic exercise training reduced the expression of CD36 mRNA by 50% compared with the levels measured in cells from the sedentary mice, in agreement with oil red lesion staining results. The mRNA levels of IL-1β and MCP-1 in the BMDM were also reduced in the exercised LDLr^–/–^ mice (46 and 36%, respectively) when compared with the cells obtained from the sedentary LDLr^–/–^ mice, in agreement with the immunostained aortic lesions. The expression of other classical proinflammatory genes, IL-6 and TNF-α, was also reduced by one-half in the BMDM of the exercised mice. The mRNA level of the cytosolic form of superoxide dismutase (SOD1), an enzyme indicative of oxidative stress, was decreased by 45% in the BMDM from the exercised mice. This finding suggests a lower ROS production in the cells of the exercised LDLr^–/–^ mice, in agreement with the results of the aortic lesions (DHE and 3-NT levels). We also observed a reduction in the expression of GADD153 mRNA, although it was not statistically significant. An essential component of macrophage motility machinery is the protein CDC42, which induces filopodia formation. We observed that CDC42 mRNA expression was decreased by 50% in the BMDM from the exercised LDLr^–/–^ mice, in agreement with previous BMDM motility results.

### Bone Marrow From the Exercised Donor Mice Reduces Atherosclerosis When Transplanted Into the Sedentary Mice

**FIGURE 6:**
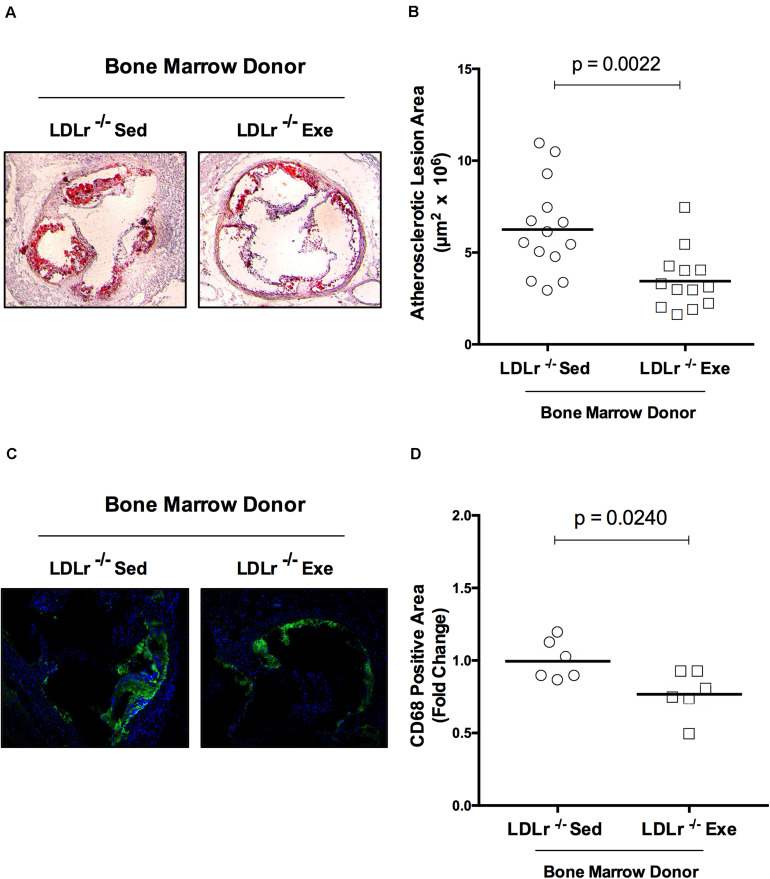
Bone marrow from exercised donor mice reduces atherosclerosis in recipient sedentary mice. Sedentary LDLr^–/–^ female mice (recipients) were transplanted with bone marrow from sedentary (Sed) or exercised (Exe) LDLr^–/–^ male mice (donors) fed a high-fat and high-cholesterol diet for 8 weeks. Representative images **(A)** and lesion area stained with oil red O, *n* = 13–14 **(B)**. The immunofluorescence images **(C)** and areas positive for macrophage CD68 (green), *n* = 6 **(D)**. Blue color represents nuclei stained with DAPI. Data are presented as the means and individual determinations, *P*-values according to the Student’s *t*-test.

The changes observed in BMDM reflect phenomena that occur in stem cells (bone marrow) and persist throughout the macrophage differentiation process *ex vivo*. Thus, we hypothesized that bone marrow transplantation from the exercised donor mice would be able to reduce atherosclerosis in sedentary recipient mice. For these experiments we used female LDLr^–/–^ as bone marrow recipient mice because they develop more severe atherosclerosis than males. The male bone marrow donor mice were exercised for 16 weeks to test long-term exercise training. Indeed, LDLr^–/–^ female sedentary mice that received bone marrow from exercised LDLr^–/–^ mice developed significantly smaller lesions than the mice transplanted with bone marrow from sedentary LDLr^–/–^ mice (Figures 6A,B). Importantly, the macrophage content of the atherosclerotic plaques was also markedly reduced in the mice that received bone marrow from the exercised donors, compared to that in the mice transplanted with bone marrow from sedentary donors (Figures 6C,D). Plasma lipid levels were similar between the transplanted groups; however, the concentration of IL-6 was diminished by 37% (*p* = 0.05) while TNF-α plasma levels showed a trend to decrease (43%, *p* = 0.07) in the group that received bone marrow from the exercised mice (Table 3). The body and tissue weights of both graft recipient groups were similar independent of the source of bone marrow (Supplementary Table 3).

**TABLE 3:** Plasma lipids and cytokines of the transplanted sedentary LDLr^–/–^ mice that received bone marrow from sedentary (LDLr^–/–^Sed) or exercised (LDLr^–/–^Exe) donor mice and fed a high-fat and high-cholesterol diet for 8 weeks.

	Bone marrow donors	
	LDLr^–/–^ Sed	LDLr^–/–^ Exe
Triglycerides (mg/dL)	257.8 ± 33.9 (10)	232.0 ± 16.3 (8)
Total cholesterol (mg/dL)	1056.3 ± 39.6 (9)	1029.9 ± 95.7 (9)
**Cytokines**		
Interleukin-10 (pg/mL)	16.0 ± 0.85 (8)	15.8 ± 0.61 (8)
Interleukin-6 (pg/mL)	7.07 ± 0.41 (6)	4.44 ± 0.99 (8)*
Tumor necrosis factor-α (pg/mL)	26.4 ± 4.7 (4)	14.9 ± 2.6 (4)^#^

Data are the mean ± SE (n). Student’s t-test: *p = 0.05, ^#^p = 0.07.

## Discussion

The beneficial adaptive responses to regular physical exercise are appreciated and include improvements in metabolism, redox state and inflammation scenario in several tissues. In the present work, we focused on macrophage phenotype and functions that are linked to arterial wall lesion development. We showed that aerobic exercise training promoted a reduction in lipid accumulation and the attenuation of the nitro-oxidative damage and inflammatory state of atherosclerotic lesions. The main and novel findings reveal that physical exercise targets both monocyte-macrophages found in the arterial wall and hematopoietic precursor cells that are relevant for the vascular wall response to atherogenic insults. Macrophages from exercise-trained mice showed an attenuation of their polarization to the inflammatory phenotype and migratory function, which was evidenced by the inhibition of the proinflammatory gene expression profile and decreased cell migration and cytoskeleton remodeling in response to chemotactic MCP-1. More importantly, these findings were associated with epigenetic mechanisms as indicated by the bone marrow from the exercised mice diminishing atherosclerosis in the recipient sedentary mice.

Several mechanisms may be raised to explain the connection between exercise and reduced macrophage activation and migration. The dysfunction of vascular endothelial layer is critically implicated in the activation and recruitment of macrophages in atherosclerosis prone conditions. When exposed to oxidized lipoproteins or cytokines, endothelial cells respond exhibiting a proinflammatory endothelial phenotype, expressing leukocyte adhesion molecules and secreting chemokines, such as MCP-1 among others. The influx of T-cells and monocytes/macrophages, contributing their own set of cytokines, creates a complex paracrine milieu of cytokines, growth factors and ROS within the vessel wall, which perpetuates a chronic pro-inflammatory state and fosters atherosclerotic lesion progression (Gimbrone and García-Cardeña, 2016). Improvement of endothelial function in atherosclerosis model reduces T-cells, macrophages and monocytes infiltration into plaques of ApoE^–/–^ mice (Schmidt et al., 2010). It is well-established that exercise training induces vasoprotective endothelial phenotype triggered by hemodynamics processes such as shear stress and mechanical stimulus (Harrison et al., 2006) and consequently, may decrease macrophage recruitment and activation. Exercise induced-laminar shear stress promotes eNOS activity and expression, increasing NO bioavailability and decreasing ROS production (Harrison et al., 2006; Szostak and Laurant, 2011), thus preserving endothelial layer integrity and suppressing chemotaxis. Another theoretical possibility is that the exercise increased blood flow could also directly influence mechanoreceptors in the circulating leukocytes decreasing their activation and subsequent transendothelial migration. Sympathetic activity to the bone marrow, elicited by physical exercise, is an additional possibility by which exercise may change the mobilization of hematopoietic stem and progenitors cells (HSPC) into peripheral circulation and subsequently change the number and function of leukocytes. However, these effects are highly dependent on the intensity of the exercise and may be transient (Emmons et al., 2016; Kröpfl et al., 2020). Finally, exercise training improves skeletal muscle secretion of myokines, extracellular cargo vesicles and metabolites that facilitates regulatory crosstalk with other tissues, including cells in the vascular wall (McGee and Hargreaves, 2020).

Some of the expected systemic effects of exercise training were observed in exercised LDLr^–/–^ mice. Although total plasma cholesterol levels did not change significantly, exercise training increased the levels of HDL cholesterol plasma fraction, in agreement with previous findings (Wang and Xu, 2017; Cai et al., 2018; Lemes et al., 2018; Ruiz-Ramie et al., 2019). HDL has several anti-atherogenic functions, such as macrophage cholesterol efflux and vascular antioxidant and anti-inflammatory functions (Camont et al., 2011). Exercise training also reduced the circulating levels of the proinflammatory cytokines TNF-α, IL-1β, and IL-6 in the LDLr^–/–^ mice, in agreement with previous findings in animal models (Ohta et al., 2005; Fukao et al., 2010), in patients with cardiac disease (Schumacher et al., 2006) or with metabolic syndrome or obesity (Pitsavos et al., 2005; Bruun et al., 2006). The prominent role of IL-1β in atherosclerosis is evidenced by the results of IL-1β gene ablation in ApoE^–/–^ mice (Kirii et al., 2003) and by neutralizing antibody therapy against IL-1β that decreased the rate of recurrent cardiovascular events, as shown in the CANTOS study (Ridker et al., 2017).

Oxidative and nitro-oxidative stress are involved in the initiation and propagation of atherosclerosis by influencing inflammatory and chemotactic cell responses (Marchio et al., 2019). Decreased ROS production was found in the arterial wall of the exercised LDLr^–/–^ mice in this study, as indicated by the lower level of fluorescent DHE-derived oxidation products. The level of nitrotyrosine, a major biomarker of protein oxidative damage caused by peroxynitrite, was also reduced in the exercised LDLr^–/–^ mouse aortic plaques. Nitrotyrosine has been related to plaque instability (Turko and Murad, 2002) and detected in human serum and atherosclerotic lesions (Pennathur et al., 2004; Upmacis, 2008). One possible explanation for these findings is that exercise, through promoting laminar shear stress, down-regulates endothelial angiotensin II type 1 receptor (AT1R) expression, leading to decreases in NADPH oxidase activity and superoxide anion production, which in turn preserves endothelial NO bioavailability and decreases oxidative/nitroxidative stress (Szostak and Laurant, 2011). Exercise training also improves tissue levels of antioxidant enzymes, such as catalase and GSH peroxidase (Takeshita et al., 2000; Linke et al., 2005) and markedly increases extracellular isoform of superoxide dismutase (Fukai et al., 2000). Structural and functional integrity of mitochondria is important for cell metabolic and redox homeostasis. Increased number and oxidative capacity of mitochondria in skeletal muscles is a well-documented effect of regular physical exercise. In addition, Kim et al. (2014) showed that exercise mediated wall shear stress increases mitochondrial biogenesis in vascular endothelium. Furthermore, 6 months of aerobic exercise training alleviates the release of endothelial microparticles (a sign of activated or apoptotic endothelial cell) in prehypertensive individuals via shear stress-induced mitochondrial biogenesis (Kim et al., 2015).

Hyperlipidemia and oxidative stress, among a large variety of stresses, trigger perturbations of ER homeostasis (Cullinan and Diehl, 2006) that may cause downstream maladaptive responses such as inflammation and cell death. It has been previously proposed that ER stress is involved in endothelial dysfunction. Indeed, induction of ER stress in resistance and conductance arteries resulted in defective endothelium-dependent relaxation, as well as decreased eNOS phosphorylation and increased NADPH oxidase activity (Kassan et al., 2012; Galán et al., 2014). ER stress markers, including GRP78, CHOP/GADD, p-IRE-1, XBP-1, ATF-6, p-PERK, and p-elf2 were reported to be elevated in atherosclerotic aorta of ApoE^–/–^ mice (Zhou et al., 2005). Here, we showed the presence of ER stress, as evidenced by GADD expression in the arteries of sedentary high fat fed LDLr^–/–^ mice. Interestingly, GADD immunostaining was widely spread with partial colocalization with macrophages (CD68). Physical exercise training markedly suppressed the expression of this ER stress marker. This finding is in agreement with previous studies. Treadmill exercise training suppressed ER stress (PERK, IRElα, and ATF6) and ameliorated endothelial dysfunction through a PPARγ-dependent mechanism with an increase in NO bioavailability in diabetic arteries (Cheang et al., 2017). In addition, Hong et al. (2018) showed that exercise training reversed the increase in CHOP expression in the superior mesenteric arteries of ApoE^–/–^ mice and ameliorated vascular dysfunction by regulating downstream signaling pathways including eNOS, UCP-2 (oxidative stress) and caspase-1 (inflammation) activities.

Macrophages in a lesion microenvironment are heterogeneously shaped, assuming inflammation promoting or suppressing and repair functions, designated M1 or M2, respectively (Tabas and Bornfeldt, 2016). Physical exercise has been identified as an inflammation suppressor in adipose tissue (Kawanishi et al., 2010, 2013), liver (Kawanishi et al., 2012), and skeletal muscle (Leung et al., 2016) by reducing the M1/M2 macrophage ratio. Furthermore, physical exercise is effective in reducing M1 markers in isolated mouse peritoneal macrophages (Chen et al., 2010) and human monocytes (Ruffino et al., 2016). In this study, we found that the BMDM from the exercised mice exhibited decreased expression of TNF-α, IL-1β, and IL-6, markers of the M1 inflammatory macrophage phenotype. These findings offer supporting evidence suggesting that physical exercise blunts macrophage M1 polarization not only within the arterial wall but also in precursor myeloid cells. Similarly, the expression of the chemoattractant MCP-1 was repressed by the exercise training, as seen in the arterial wall and BMDM from the exercised LDLr^–/–^ mice. The pro-atherogenic role of MCP-1 and its receptor CCR2 in monocyte recruitment into the arterial wall is well established (Boring et al., 1998; Tsou et al., 2007; França et al., 2017). MCP-1 and its receptor are involved in the regulation of the cytoskeletal changes required for cell migration (Gerszten et al., 2001). In this study, we found that the BMDM migration response to MCP-1 after physical exercise training was reduced. This finding probably explains the significant reduction in macrophage content (CD68) in the atherosclerotic plaques. The macrophage become motile through sequential steps: protrusion of filopodia and lamellipodia at the leading front, adhesion of the protruding edge to the substratum, contraction of the cytoplasmic actin/myosin, and finally release from contact sites at the tail of the cell (Jones, 2000). The activation of the proteins CDC42 and RAC leads to the formation of filopodia and lamellipodia, respectively. RAC-1 is localized at the leading edge of motile cells in response to a chemoattractant stimulus. Accordingly, we found no such localization of RAC-1 in macrophages from the exercised mice in response to MCP-1. Furthermore, CDC42 mRNA was markedly decreased in the BMDM of the exercised mice. Endothelial-specific deletion of CDC42 attenuates chronic inflammation and plaque formation in atherosclerotic mice (Ito et al., 2014).

Growing evidence has revealed that unresolving atherosclerotic inflammation is associated with immunological memory. Known as trained immunity, this effect is also established in bone marrow precursor cells (Zhong et al., 2020). For instance, hematopoietic stem/progenitor cells (HSPCs) conditioned by hypercholesterolemia aggravate atherosclerosis after they have been grafted into a normocholesterolemic microenvironment (Seijkens et al., 2014). In addition, LDLr^–/–^ mice fed a chow diet showed enhanced atherosclerosis after receiving bone marrow from mice fed a high-fat western-type diet (van Kampen et al., 2014). In contrast, physical exercise appears to promote a beneficial effect on immunological memory. A recent study revealed that exercise training decreased the proliferation of HSPCs and circulating leukocytes, impaired monocyte differentiation into macrophages and attenuated atherosclerosis in mice (Frodermann et al., 2019). Here, we show that aerobic exercise training promoted a differential macrophage phenotype and function (cytokine expression, migration and motility) that persisted in myeloid-derived macrophages, leading to sedentary recipients of bone marrow to slowing down the development lipid laden and less inflamed atherosclerotic lesions. A limitation of this study design is that we cannot assure that physical exercise, *per se*, would be able to induce these epigenetic anti-atherogenic effects because donor mice received high fat diet concomitantly with exercise training. However, since many harmful metabolic and redox epigenetic effects of high fat diets are well known, we may hypothesize that exercise training counteracted or reversed these diet detrimental effects.

## Conclusion

Collectively, these data demonstrate that aerobic exercise training slows down the progression of atherosclerotic lesion and positively alters plaque feature and inflammation profile. These findings are associated with exercise-induced inhibition of activation and recruitment of bone marrow-derived macrophages. Thus, we propose that regular aerobic exercise induces epigenetic anti-inflammatory changes in bone marrow stem cells, attenuating atherosclerosis.

## Data Availability Statement

The raw data supporting the conclusions of this article will be made available by the authors, without undue reservation.

## Ethics Statement

The animal study was reviewed and approved by the State University of Campinas Committee for Ethics in Animal Experimentation (CEUA/UNICAMP).

## Author Contributions

TR and AW: study design, data acquisition, analyses and interpretation, and manuscript writing; LC, EL-G, JS, and RS: data acquisition and analyses and interpretation. HO: project conception, study design, data analyses, and manuscript writing. All authors contributed to the article and approved its final version.

## Conflict of Interest

The authors declare that the research was conducted in the absence of any commercial or financial relationships that could be construed as a potential conflict of interest.

## Supplementary Material

Supplementary Figure 1 Training parameters before and after the exercise training program.

Supplementary Table 1 Experimental diets composition.

Supplementary Table 2 Oligonucleotides used for determining the gene expression profile in BMBM by RT-PCR.

Supplementary Table 3 Body and tissue weights of transplanted sedentary LDLr^–/–^ mice that received bone marrow from sedentary (LDLr^–/–^Sed) or exercised (LDLr^–/–^Exe) donor mice and were fed a high-fat and high-cholesterol diet for 8 weeks.
