# Supplementary figures and images for: The Anti-atherogenic Role of Exercise Is Associated With the Attenuation of Bone Marrow-Derived Macrophage Activation and Migration in Hypercholesterolemic Mice
Source: Front Physiol. 2020 Nov 23;11:599379. doi: 10.3389/fphys.2020.599379 (PMC7719785; doi:10.3389/fphys.2020.599379)

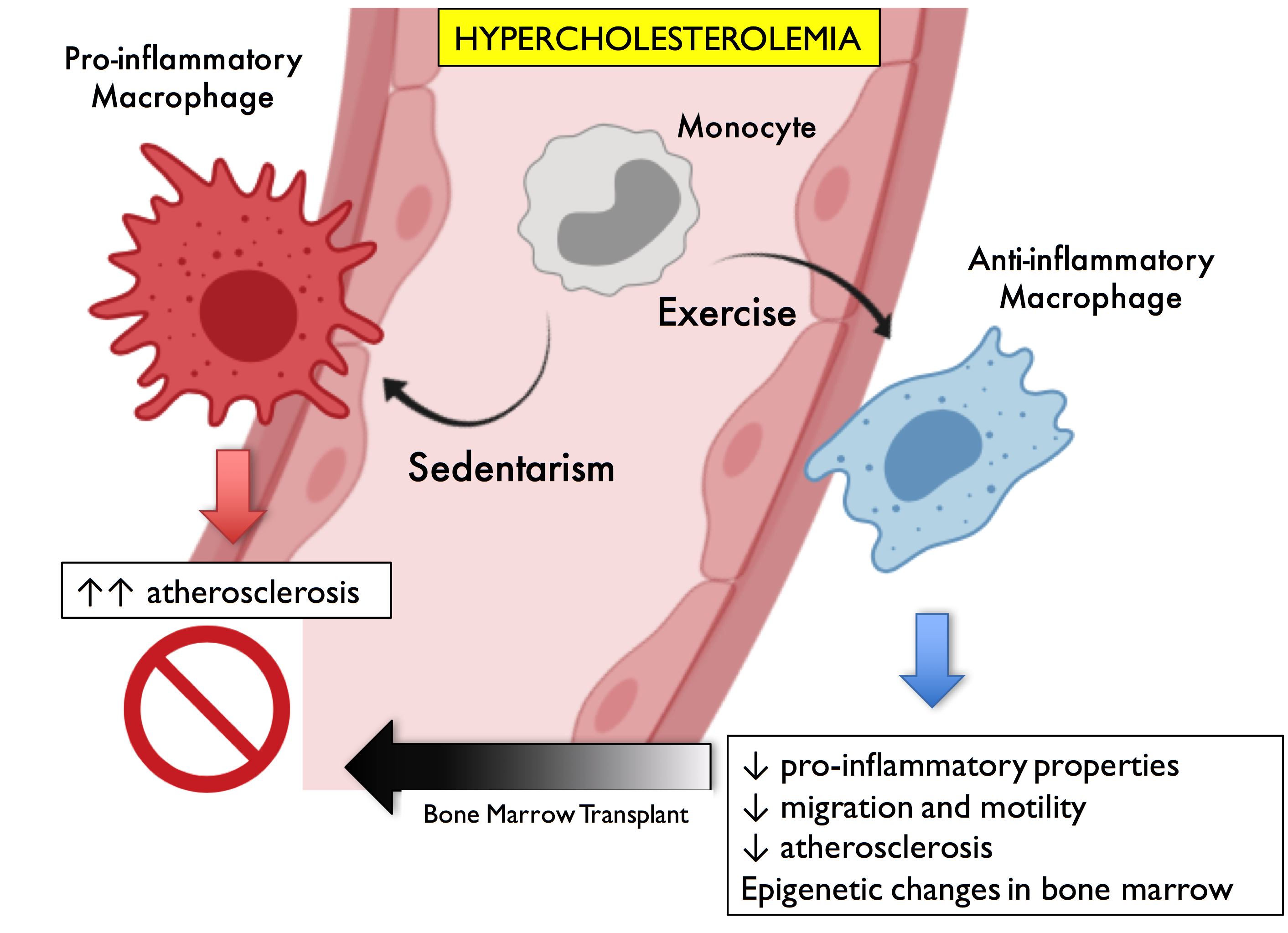

Supplement: Supplementary Figure 1 — Training parameters before and after the exercise training program. [file Image_1.TIFF]
