# Supplementary material for: The Anti-atherogenic Role of Exercise Is Associated With the Attenuation of Bone Marrow-Derived Macrophage Activation and Migration in Hypercholesterolemic Mice
Source: Front Physiol. 2020 Nov 23;11:599379. doi: 10.3389/fphys.2020.599379 (PMC7719785; doi:10.3389/fphys.2020.599379)
Supplement: Supplementary Table 1 — Experimental diets composition. [file Data_Sheet_1.PDF]

Anti-atherogenic role of exercise is associated with attenuation of bone marrow derived macrophage activation and migration in hypercholesterolemic mice.

Thiago Rentz, Amarylis C. B. A. Wanschel, Leonardo de Carvalho Moi, Estela Lorza-Gil, Jane C. de Souza, Renata R. dos Santos, Helena C. F. Oliveira.

SUPPLEMENTARY MATERIAL

Supplementary Table 1. Composition of the Experimental Diets

Components (g)	AIN-93M	High Fat Diet	High fat and high cholesterol diet
Casein	140	140	195
Corn Starch	465.7	208.7	50
Dextrin	155	100	100
Sucrose	100	100	341
Milk Fat	0	312	200
Soybean/corn Oil	40	40	10*
Cellulose	50	50	50
Mineral Mix	35	35	35
Vitamin Mix	10	10	10
DL-Methionine	3	3	3
L-Cysteine	1.8	1.8	3
Calcium Carbonate	4	4	4
Choline Bitartrate	2.5	2.5	2
Butylhydroquinone	0.01	0.01	0
Cholesterol	0	0	1.5
**Total**	**1000**	**1000**	**1000**

* Corn Oil. AIN - American Institute of Nutrition.

Supplementary Table 2. Oligonucleotides used for determining gene expression profile by RT-PCR in bone marrow derived macrophages (BMBM)

**Genes**	**Primers**	
GAPDH	forward<br>reverse	5` CCTGCACCACCAACTGCT 3`<br>5` GCCCCACGGCCATCACGC 3`
CD36	forward<br>reverse	5` GGAACTGTGGGCTCATTGC 3`<br>5` CATGAGAATGCCTCCAAACAC 3`
IL-1β	forward<br>reverse	5`ACAAGGAGAGACAAGCAACGAC 3`<br>5` TCTTCTTTGGGTATTGTTTGGG 3`
MCP1/CCL2	forward<br>reverse	5`GCTGGAGAGCTACAAGAGGATCA 3`<br>5` ACAGACCTCTCTCTTGAGCTTGGT 3`
IL-6	forward<br>reverse	5` CACGGCCTTCCCTACTTCAC 3`<br>5`GGTCTGTTGGGAGTGGTATC 3`
TNF-α	forward<br>reverse	5` CCCTCCTGGCCAACGGCATG 3`<br>5` TCGGGGCAGCCTTGTCCCTT 3`
SOD1	forward<br>reverse	5'AACCAGTTGTGTTGTCAGGAC 3'<br>5'CCACCATGTTTCTTAGAGTGAGG 3'
CHOP/GADD153	forward<br>reverse	5'CTGGAAGCCTGGTATGAGGAT 3'<br>5'CAGGGTCAAGAGTAGTGAAGGT 3'
CDC42	forward<br>reverse	5`ACGTGTCCCCACCTGGTGCT 3`<br>5` GCGAGACAGCGTCCACCCAC 3`

Supplementary Table 3. Body and tissue weights of sedentary recipient LDLr$^{-/-}$ mice that were transplanted with bone marrow from sedentary or exercised LDLr$^{-/-}$ donor mice after 8 weeks on a high fat and high cholesterol diet.

	**Bone Marrow Donor**	
	**Ldlr$^{-/-}$ Sed**	**Ldlr$^{-/-}$ Exe**
Body Weight (g)	22.3 ± 0.5 (14)	22.2 ± 0.5 (13)
Epididymal Fat Pad (g/100 g)	0.237 ± 0.03 (14)	0.295 ± 0.03 (13)
Liver (g/100g)	0.991 ± 0.03 (14)	1.037 ± 0.03 (13)
Gastrocnemius Muscle (g/100 g)	0.121 ± 0.006 (14)	0.134 ± 0.008 (13)
Spleen (g/100 g)	0.060 ± 0.004 (14)	0.066 ± 0.004 (13)

Data are mean ± SE (n). Student's t test.

Supplementary Figure 1

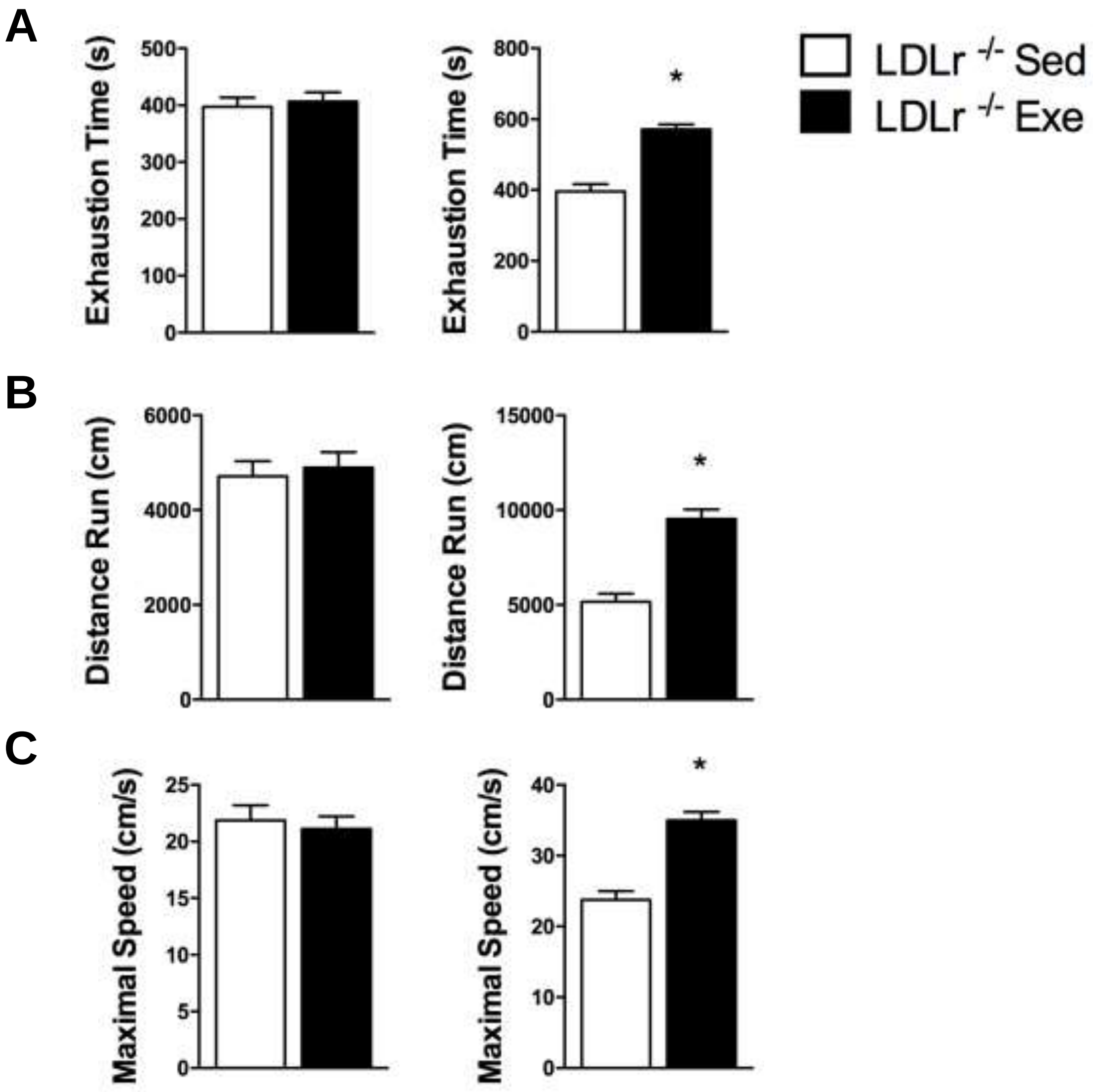

Supplementary Figure 1 – Training related parameters before and after the exercise training program. Maximal exercise capacity test before (**left graphs**) and after (**right graphs**) exercise training program. **A**) Exhaustion time, **B**) distance run and **C**) maximal speed reached. Data are mean ± SE, n=8 for sedentary (Sed) mice and n=9 for exercised (Exe), *$p<0.05$, Student's t test.
